# Overexpression of PSAT1 regulated by G9A sustains cell proliferation in colorectal cancer

**DOI:** 10.1038/s41392-020-0147-5

**Published:** 2020-04-17

**Authors:** Huijuan Wang, Longzhen Cui, Dandan Li, Ming Fan, Zhangnan Liu, Chunqi Liu, Sijing Pan, Lei Zhang, Hailong Zhang, Yinglan Zhao

**Affiliations:** 1 Joint National Laboratory for Antibody Drug Engineering, Key Laboratory of Cellular and Molecular Immunology of Henan Province, Institute of Translational Medicine, School of Basic Medicine, Henan University, Kaifeng, 475004 China; 2 Translational Medicine Center, Huaihe Hospital of Henan University, Kaifeng, 475000 China; 3 Department of Pharmacy, West China Hospital of Stomatology, Sichuan University, Chengdu, 610041 China; 4 State Key Laboratory of Biotherapy and Cancer Center, West China Hospital, West China Medical School and Collaborative Innovation Center for Biotherapy, Sichuan University, Chengdu, 610041 China

**Keywords:** Cancer metabolism, Cancer

**Dear Editor**,

Colorectal cancer (CRC) is one of the most common cancers that contributes to cancer morbidity and mortality according to the National Cancer Institute’s report. The standard of care is still surgical resection and neoadjuvant chemoradiation therapy, which may result in serious effects on quality of life in patients.^1^ Currently, many efforts have been aimed at precision medicine in CRC, which highlights the urgent need to identify accurate biomarkers for diagnosis and treatment that can be translated into clinical use.^2^ As an important precursor for biomolecule synthesis, serine plays an essential role in cell proliferation. Recently, the serine synthesis pathway (SSP) has been shown to be activated during the pathogenesis of many cancers.^3^ Phosphoserine aminotransferase (PSAT1), the enzyme that catalyzes the second step of the SSP, has been shown to correlate with cell proliferation and cancer development.^4^ Overexpression of PSAT1 was found in non-small cell lung cancer, breast cancer, and esophageal squamous cell carcinoma and was shown to enhance tumorigenesis and metastasis.^5^ These studies suggested that PSAT1 could play a role as a proproliferative and prosurvival factor in the process of carcinogenesis. However, less is known about the expression of PSAT1 and the underlying mechanism in CRC, which prompted us to explore its role and regulatory mechanism in the initiation and development of CRC.

Based on our previous study,^6^ the SSP was activated in CRC (Supplementary Fig. S1). First, we analyzed the relative mRNA expression levels of the related metabolic enzymes in 12 CRC cancer tissue specimens and their adjacent matched normal colorectal tissues by quantitative real time polymerase chain reaction. The mRNA levels of PSAT1 and serine hydroxymethyltransferase (SHMT1) were significantly increased (*p* < 0.05) in CRC tumor tissues compared with the corresponding normal controls (Supplementary Fig. S2a). To further investigate the aberrant expression, we then compared their expression with colon and rectal cancer mRNA-Seq data from The Cancer Genome Atlas (TCGA). PSAT1 was aberrantly upregulated (Fig. 1a), but SHMT1 was not (Supplementary Fig. S2b). Next, the difference in PSAT1 protein expression levels between CRC tumor tissues and normal controls was investigated using western blotting (Fig. 1b) and immunohistochemistry on tissue microarrays (Fig. 1c). Overall, these results indicated that PSAT1 was aberrantly activated in CRC and implied that it might play an important role in the development of CRC.

**Fig. 1:**
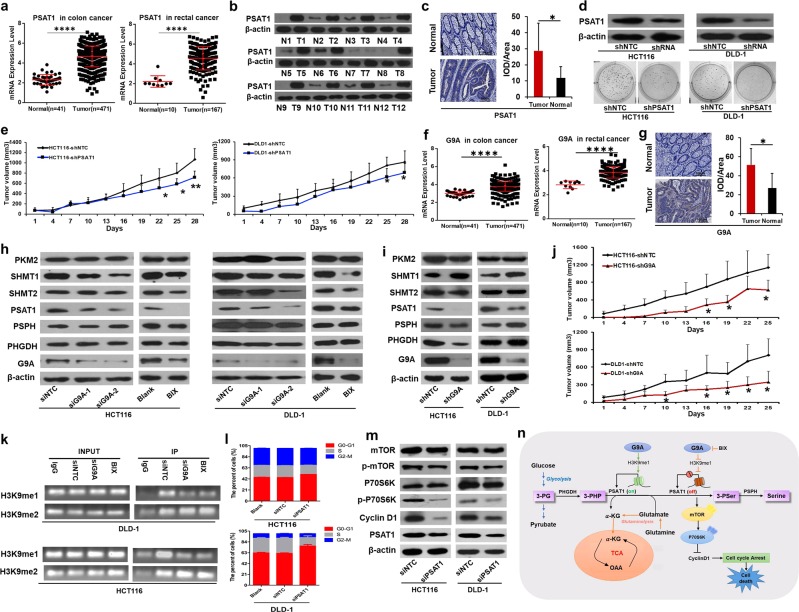
PSAT1 was regulated by G9A and enhanced cell proliferation in colorectal cancer. **a** Relative expression of PSAT1 in normal and tumor tissues from colon cancer and rectal cancer samples from the TCGA database (FC (PSAT1 in colon cancer) = 2.02; FC (PSAT1 in rectal cancer) = 2.06; ****p < 0.0001; cancer versus normal). **b** PSAT1 expression between CRC tissue specimens and the corresponding normal specimens was examined by western blot assay (*n* = 12 pairs; N normal, T tumor). **c** Representative immunohistochemical images and semiquantitative analysis of PSAT1 protein between CRC tissue specimens and the corresponding normal tissues in the tissue chip (immunohistochemical staining, scale bar = 100 µm, *n* = 30 pairs, *p < 0.05). **d** Colony formation assay of HCT116 and DLD-1 cells (stably expressing PSAT1 shRNA) in soft agar for 14 days. **e** Xenograft tumor volumes were determined in nude mice after generation of tumors using HCT116 and DLD-1 cells stably expressing NTC or PSAT1 shRNA. (*n* = 5, *p < 0.05, **p < 0.01). **f** Relative expression of G9A in the normal and cancer samples of CRC from the TCGA database. The fold changes (FCs) of G9A expression in colon and rectal cancer were 1.26 and 1.37, respectively (****p < 0.0001; cancer versus normal). **g** Representative immunohistochemical images and semiquantitative analysis of G9A protein between CRC tissue specimens and the corresponding normal specimens in the tissue chip immunohistochemical staining; scale bar = 100 µm; *n* = 30 pairs; *p < 0.05). **h**, **i** After depletion of G9A, the protein expression of G9A and related metabolic enzymes in HCT116 and DLD-1 cells was investigated by western blot assay. **j** Xenograft tumor volumes were determined in nude mice after generation of tumors using HCT116 and DLD-1 cells stably expressing NTC or G9A shRNA (*n* = 5, *p < 0.05). **k** NTC or G9A siRNA was transfected into DLD-1 and HCT116 cells or BIX (BIX01294, 5 μM) was added to DLD-1 and HCT116 cells for 48 h. H3K9me1 and H3K9me2 levels in the PSAT1 promoter were analyzed by ChIP assay. **l** Cell cycle analyses were carried out in HCT116 and DLD-1 cells expressing NTC or PSAT1 siRNA by flow cytometry. **m** The protein expression levels of total mTOR, p-mTOR, total P70S6K, p-P70S6K, and cyclin D1 were examined by western blot in HCT116 and DLD-1 cells expressing NTC or PSAT1 siRNA. **n** A model of the possible mechanism underlying PSAT1 regulation of cancer development in CRC

To investigate the potential function of PSAT1 in CRC cells, we first knocked down the level of PSAT1 in HCT116 and DLD-1 cells using transient transfection of specific siRNAs (Supplementary Fig. S3a). HCT116 and DLD-1 cells with knockdown of PSAT1 had dramatically suppressed cell viability (Supplementary Fig. S3b, c), migration (Supplementary Fig. S3d), and invasion (Supplementary Fig. S3e) compared with cells expressing the nontarget control (NTC). Serine starvation also inhibited rapid cell proliferation (Supplementary Fig. S3g). To further explore the role of PSAT1 in vivo, a xenograft experiment in nude mice was performed. The tumors derived from cells with stable knockdown of PSAT1 (Fig. 1d) resulted in a remarkably slower growth rate (Fig. 1e) and smaller tumor size (Supplementary Fig. S3f) than tumors from the cancer cells expressing the NTC. Altogether, these results suggested that PSAT1 played an important role in CRC cell proliferation and metastasis.

To explore the regulatory mechanism of PSAT1 in CRC, we focused on the effect of G9A on PSAT1 transcription. G9A, also known as EHMT2, is a histone lysine methyltransferase that catalyzes the monomethylation and dimethylation of histone H3 lysine 9 (H3K9me1 and H3K9me2, respectively) in euchromatin.^7^ Recently, more studies have indicated G9A overexpression in many types of human cancers.^8,9^ In our results, G9A expression was abnormally elevated in CRC tissues compared with normal controls (Figs. 1f, g and Supplementary Fig. S4). To investigate whether the overexpression of G9A was related to the function of PSAT1 in CRC, we depleted the expression of G9A in HCT116 and DLD-1 cells via RNA interference and the chemical inhibitor BIX (5 μM) and found that PSAT1 expression was remarkably repressed, but the other related metabolic enzymes in this pathway were not repressed (Figs. 1h, i and Supplementary Fig. S5). Cell growth, migration, and invasion were also observably inhibited in both CRC cell lines after inhibition of G9A in vitro (Supplementary Figs. S6 and S7). Moreover, knockdown of G9A also significantly inhibited cell proliferation in vivo, resulting in smaller tumor sizes (Fig. 1j). To investigate whether PSAT1 was transcriptionally regulated by G9A in CRC, we performed chromatin immunoprecipitation experiments, which revealed that silencing G9A lowered the H3K9me1 levels in the promoter region of PSAT1 (Figs. 1k and Supplementary Figs. S8 and S9). The H3K9me2 levels in the same region did not obviously change. Taken together, these results suggested that PSAT1 was directly transcriptionally activated by G9A, primarily through increases in H3K9me1 levels.

To further validate the role of PSAT1 in the proliferation of CRC cells, we silenced PSAT1 with siRNA in CRC cells; the results showed that silencing of PSAT1 obviously increased the proportion of cells in G0/G1 phase and decreased the percentage of cells in S phase (Fig. 1l). Cyclin D1, as an important regulator of G1 to S phase progression, was markedly degraded along with knockdown of PSAT1^10^ (Fig. 1m). Mammalian target of rapamycin (mTOR) has been shown to sustain cellular growth and proliferation in many cancer cell types. In our study, the phosphorylation of mTOR and S6K was markedly reduced following silencing of PSAT1, while the total mTOR and S6K levels were almost the same as those in the control cells with the nontarget siRNA (Fig. 1m). Similar changes occurred after depletion of G9A (Supplementary Fig. S10).

Overall, our work demonstrated that PSAT1 was abnormally increased by G9A transcriptional activation in CRC, which not only activated serine biosynthesis but also provided α-KG (α-ketoglutarate) for entry into the TCA (tricarboxylic acid cycle) cycle (Supplementary Fig. S11). The downregulation of PSAT1 induced the degradation of cyclin D1 through the mTOR pathway, which resulted in cell cycle arrest and cell death (Fig. 1n). Our study not only demonstrated that PSAT1 played an important role in the development of CRC but also identified a new regulatory mechanism of PSAT1 as an oncogene. These findings suggest that PSAT1 may serve as a potential therapeutic target in CRC.

## Supplementary information

Revised Supplementary Materials-2
