# Supplementary material for: Overexpression of PSAT1 regulated by G9A sustains cell proliferation in colorectal cancer
Source: Signal Transduct Target Ther. 2020 Apr 17;5:47. doi: 10.1038/s41392-020-0147-5 (PMC7162942; doi:10.1038/s41392-020-0147-5)
Supplement: Supplementary file 1 — Revised Supplementary Materials-2 [file 41392_2020_147_MOESM1_ESM.docx]

Supplementary Materials for

**Overexpression of PSAT1 regulated by G9A sustains cell proliferation in colorectal cancer**

Huijuan Wang^1^, Longzhen Cui^2^, Dandan Li^3^, Ming Fan^1^, Zhangnan Liu^1^, Chunqi Liu^4^, Sijing Pan^1^, Lei Zhang^1^, Hailong Zhang^1^*, Yinglan Zhao^4^*

^1^ Joint National Laboratory for Antibody Drug Engineering, Key Laboratory of Cellular and Molecular Immunology of Henan Province, Institute of Translational Medicine, School of Basic Medicine, Henan University, Kaifeng, 475004, China;

^2^ Translational Medicine Center, Huaihe Hospital of Henan University, Kaifeng, 475000, China

^3^ Dept. of Pharmacy, West China Hospital of Stomatology Sichuan University, Chengdu 610041, China

^4^ State Key Laboratory of Biotherapy and Cancer Center, West China Hospital, West China Medical School, and Collaborative Innovation Center for Biotherapy, Sichuan University, Chengdu 610041, China

*Correspondence e-mail: [hailong6891@163.com](mailto:hailong6891@163.com) to Hailong Zhang

[zhaoyinglan@scu.edu.cn](mailto:zhaoyinglan@scu.edu.cn) to Yinglan Zhao

**This file includes:**

Supplemental Figures

Fig. S1 The related metabolic enzymes of the serine and glycine metabolic pathway.

Fig. S2 PSAT1 was aberrantly upregulated in CRC tissues.

Fig. S3 PSAT1 promoted CRC tumor growth and metastasis.

Fig. S4 G9A was abnormally overexpressed in CRC tissues.

Fig. S5 The mRNA expression of G9A and PSAT1 in HCT116 and DLD-1 cells after depletion of G9A by siRNA or BIX.

Fig. S6 Knockdown of G9A inhibited the proliferation and metastasis of CRC cells in vitro.

Fig. S7 Depletion of G9A reduced the proliferation of CRC cells in vivo.

Fig. S8 CHIP-qPCR analysis of H3K9me1 and H3K9me2 levels at the promoters of PSAT1 in HCT116 and DLD-1 after depletion of G9A by siRNA or BIX.

Fig. S9 Levels of H3K9me1 and H3K9me2 proteins were analyzed using Western blot after depletion of G9A by siRNA or BIX.

Fig. S10 The protein expression levels of total mTOR, p-mTOR, total P70S6K and p-P70S6K were examined by western blot in HCT116 and DLD-1 cells after depletion of G9A by shRNA or BIX

Fig. S11 The mRNA levels of the TCA metabolic enzymes after depletion of G9A were examined by Real-Time PCR assay.


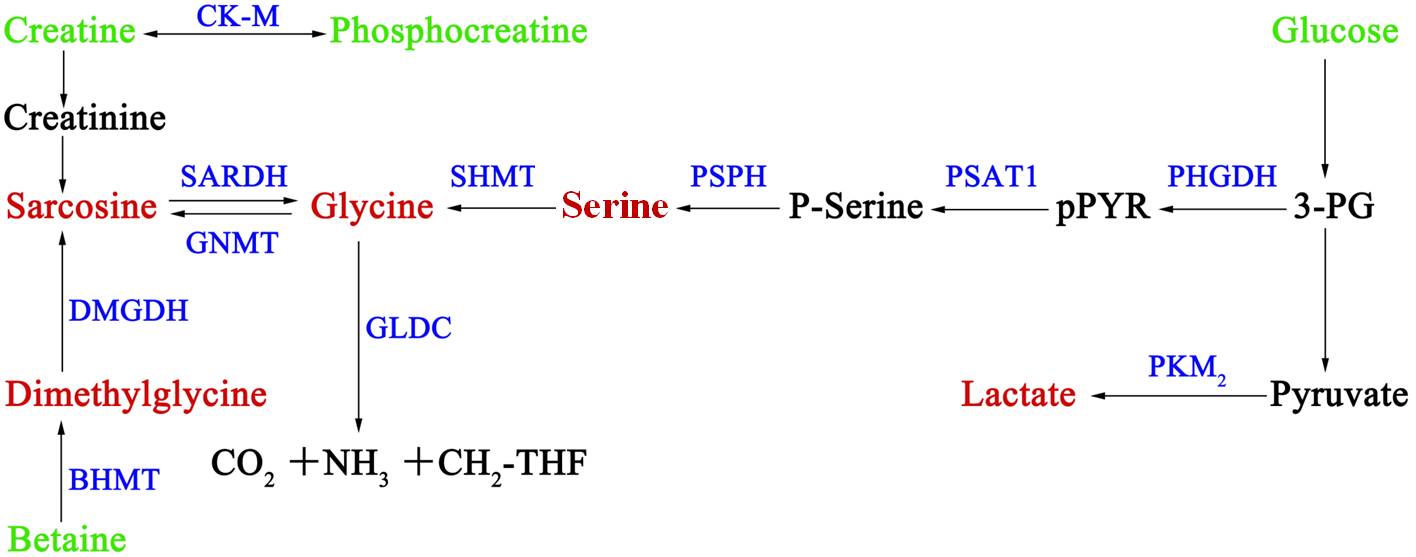


**Fig. S1 The related metabolic enzymes of the serine and glycine metabolic pathway.** Green: lower concentration in rectal cancer patients than in normal controls. Red: higher concentration in rectal cancer patients than in normal controls. Blue: the related metabolic enzymes. PKM2, Pyruvate kinase M2; PHGDH, Phosphoglycerate dehydrogenase; PSAT1, Phosphoserine aminotransferase 1; PSPH, Phosphoserine phosphatase; SHMT1/2, serine hydroxymethyltransferase I/II; GLDC, Glycine decarboxylase; GNMT, Glycine -N- methyltransferase; SARDH, sarcosine dehydrogenase; DMGDH, Dimethylglycine dehydrogenase; BHMT, Betaine homocysteine methyltransferase; CK, Creatine kinase.


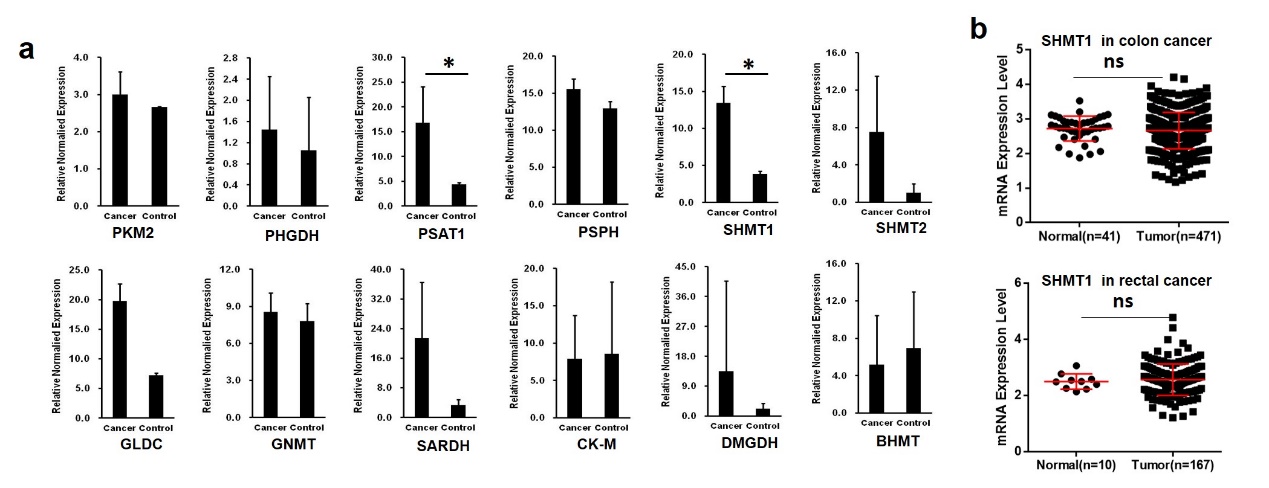


**Fig. S2** **PSAT1 was aberrantly upregulated in CRC tissues**. **a** The RNA transcription levels of the related metabolic enzymes between CRC tissue specimens and the corresponding normal specimens were examined by Real-Time PCR assay. (n=12 pairs, *p< 0.05). **b** Relative expression of SHMT1 in the normal and tumor samples of colon cancer and rectal cancer from the TCGA database.


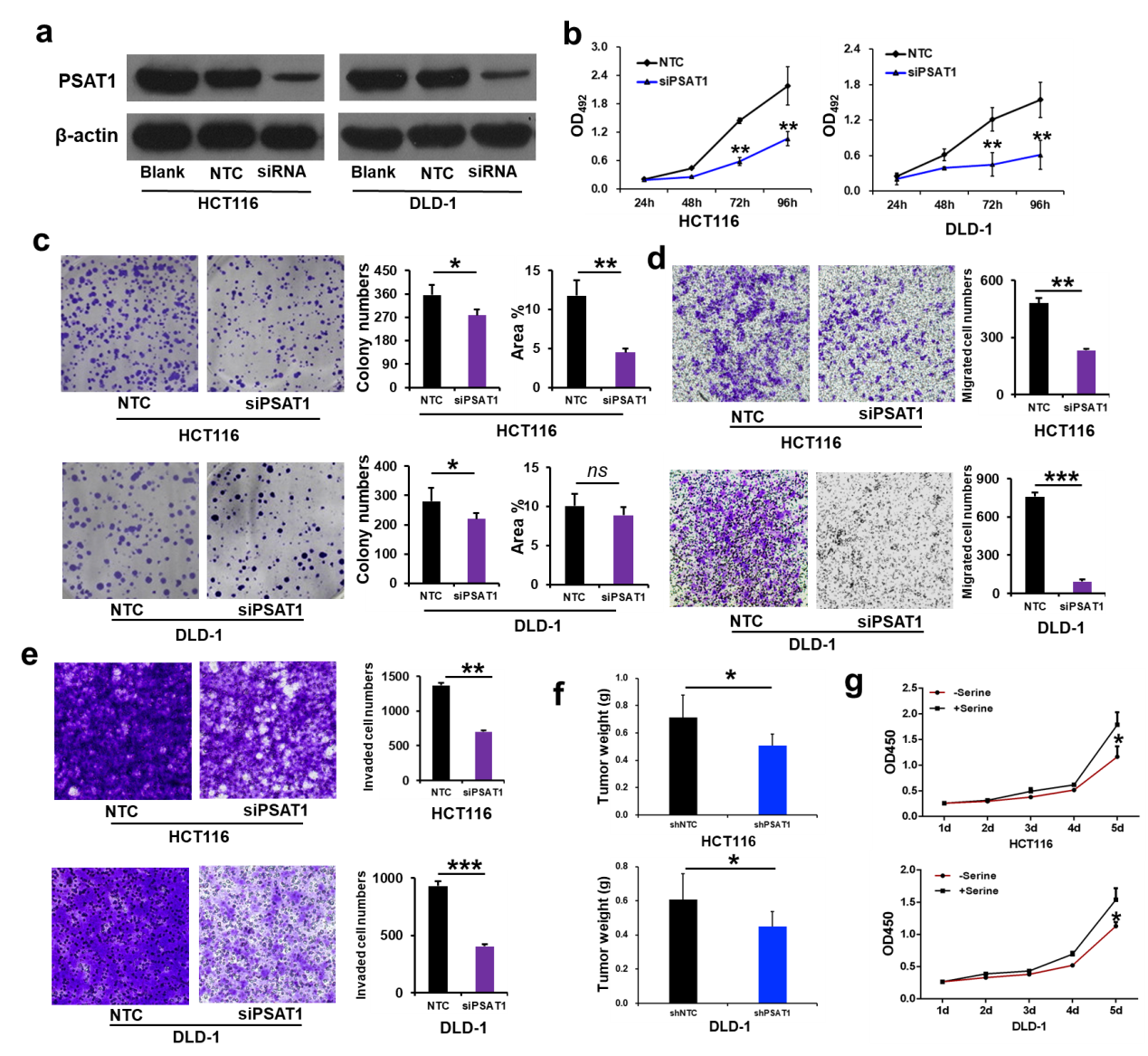


**Fig. S3** **PSAT1 promoted CRC tumor growth and metastasis. a** After being transfected with NTC or PSAT1 siRNA for 72 hours, the protein expression of PSAT1 in HCT116 and DLD-1 cells was investigated by western blot assay.  **b** Cell proliferation assay of HCT116 and DLD-1 cells expressed by NTC or PSAT1 siRNA for 72 hours using CCK-8. Error bars represented SD (n=3, **p< 0.01). **c** Colony formation assays of HCT116 and DLD-1 cells expressed of NTC or PSAT1 siRNA for 14 days. Representative photographs of cell colonies were on the left. The statistical graphs from three independent experiments were on the right. **d/e** Cell migration(**d**) and invasion (**e**) assays of HCT116 and DLD-1 cells expressed of NTC or PSAT1 siRNA. Representative photographs of cells on the membrane were on the left. The statistical graphs from three random fields were on the right. **f** The xenograft tumor weight assay was carried out in nude mice using HCT116 and DLD-1 cells stably expressing NTC or PSAT1 shRNA. Tumor weights were measured after dissection (right panel). (n=5, *p< 0.05). **g** CRC cells were grown in complete medium (containing serine) or equivalent medium lacking serine (average of triplicate wells). The proliferation assays were performed using CCK-8.


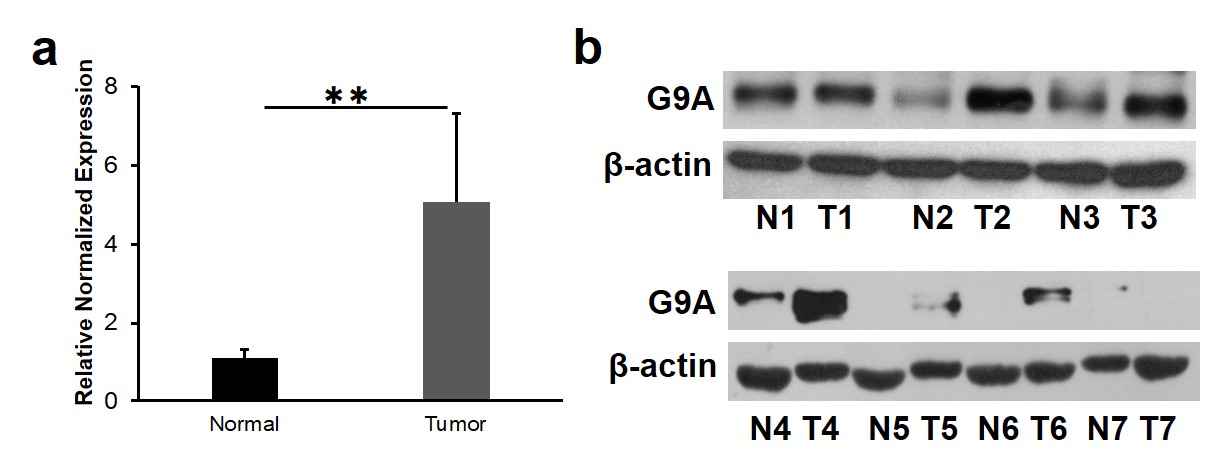


**Fig. S4 G9A was abnormally overexpressed in CRC tissues.** **a** The RNA transcription level of G9A between CRC tissue specimens and the corresponding normal specimens was examined by Real-Time PCR assay. (n=7 pairs, **p< 0.01). **b** G9A expression between CRC tissue specimens and the corresponding normal specimens was examined by western blot assay.


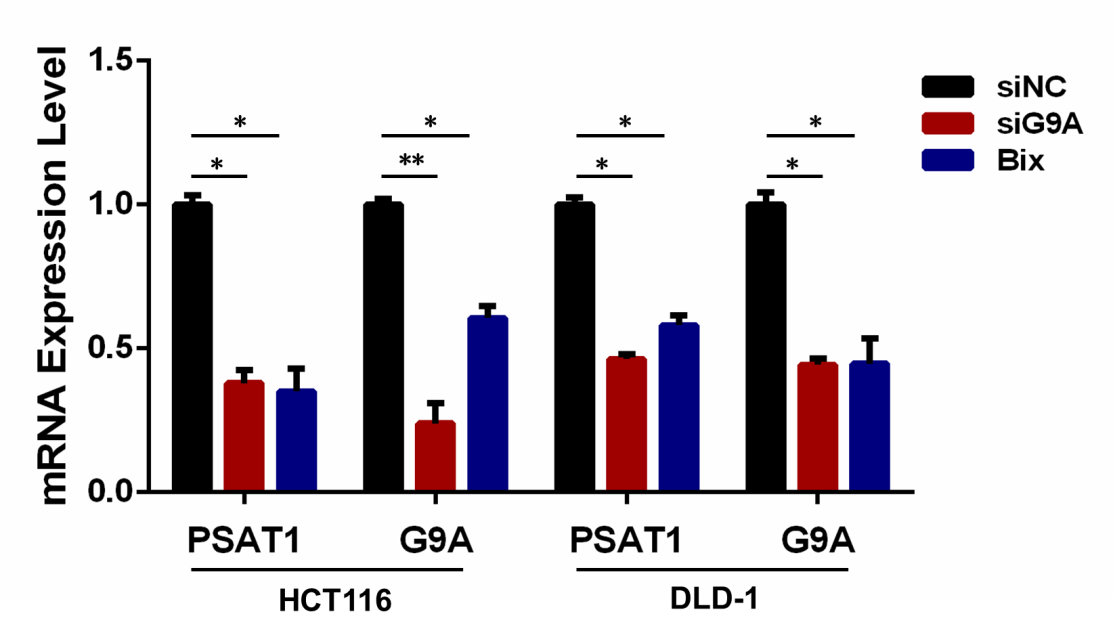


**Fig. S5 The mRNA expression of G9A and PSAT1 in HCT116 and DLD-1 cells after depletion of G9A by siRNA or BIX.**


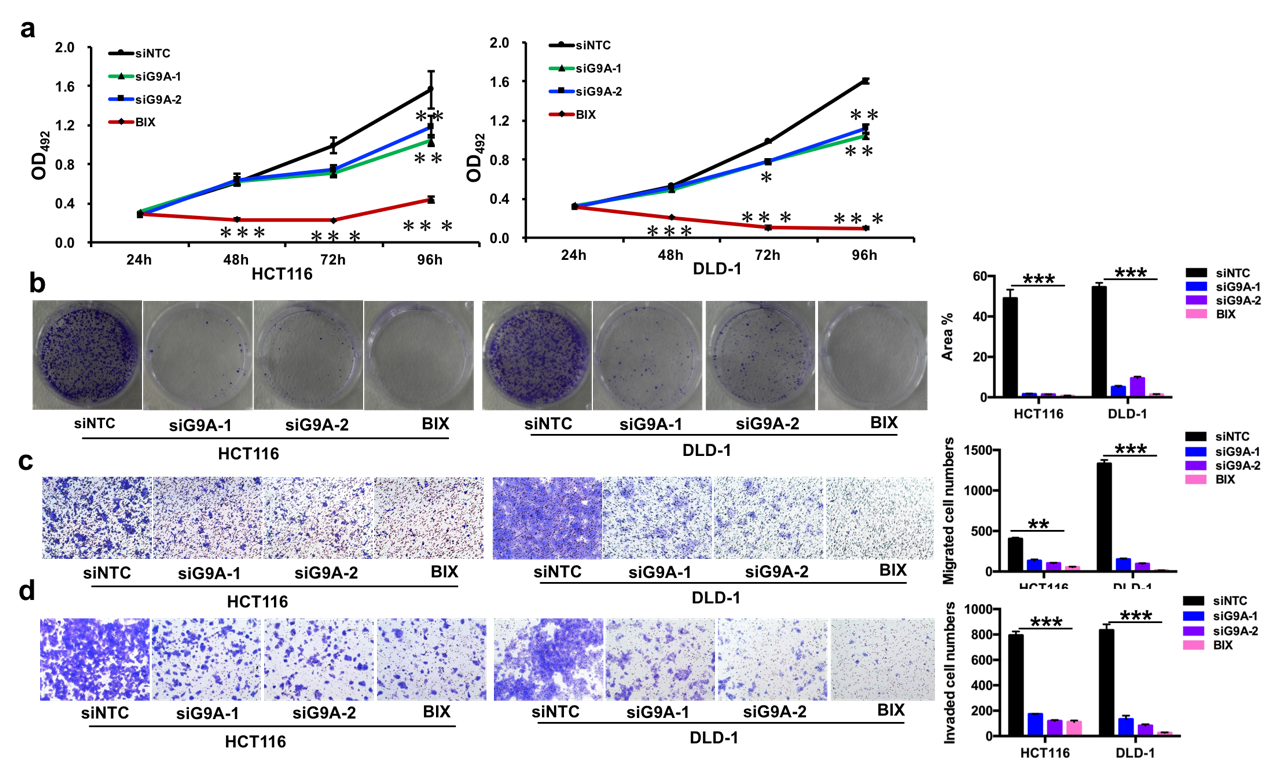


**Fig. S6 Knockdown of G9A inhibited the proliferation and metastasis of CRC cells in vitro. a** Cell proliferation assay of HCT116 and DLD-1 cells expressed of NTC or G9A siRNA, and with 5μM BIX for 72 hours using CCK-8. Error bars represent SD (n=3, *p< 0.05, **p< 0.01, ***p< 0.001). **b** Colony formation assays of HCT116 and DLD-1 cells expressed of NTC or G9A siRNA, and treated with 5μM BIX for 10 days. Representative photographs of cell colonies were on the left. The statistical graphs from three independent experiments were on the right. **c/d** Cell migration (**c**) and invasion (**d**) assays of HCT116 and DLD-1 cells expressed of NTC or G9A siRNA, and treated with 5μM BIX. Representative photographs of cells on the membrane were on the left. The statistical graphs from three random fields were on the right.


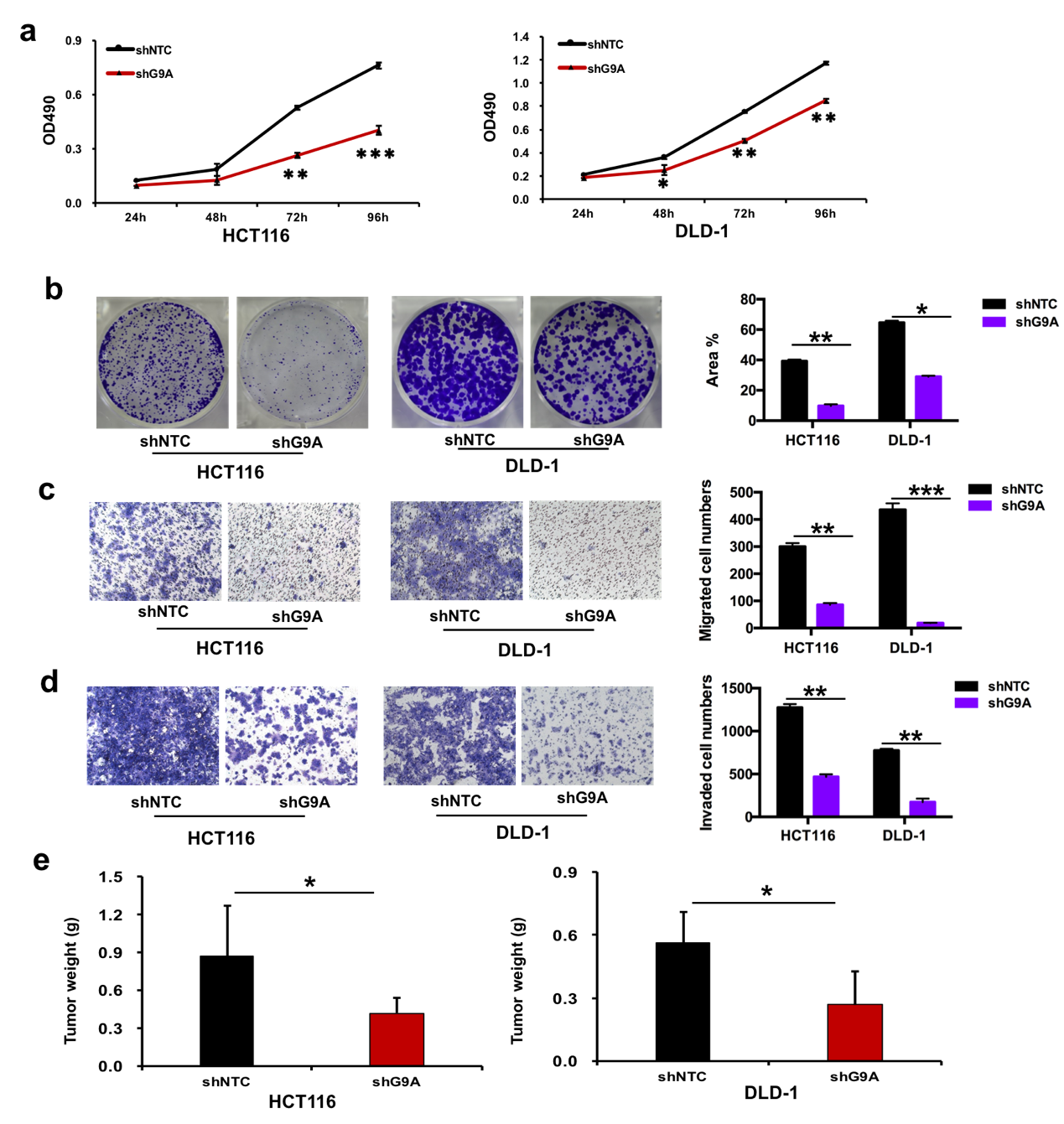


**Fig. S7 Depletion of G9A reduced the proliferation of CRC cells in vivo. a** Cell proliferation assay was carried out in HCT116 and DLD-1 cells stably expressed of NTC or G9A shRNA for 72 hours using CCK-8. Error bars represented SD (n=3, *p< 0.05, **p< 0.01, ***p< 0.001). **b** Colony formation assay was carried out in HCT116 and DLD-1 cells stably expressed of NTC or G9A shRNA for 14 days. Representative photographs of cell colonies were on the left. The statistical graphs from three independent experiments were on the right. (**c**) and invasion (**d**) assays were carried out in HCT116 and DLD-1 cells stably expressed of NTC or G9A shRNA. Representative photographs of cells on the membrane were on the left. The statistical graphs from three random fields were on the right. **e** The xenograft tumor weight assay was carried out in nude mice using HCT116 and DLD-1 cells stably expressing of NTC or G9A shRNA. Tumor weights were measured after dissection (right panel). (n=5, *p< 0.05).


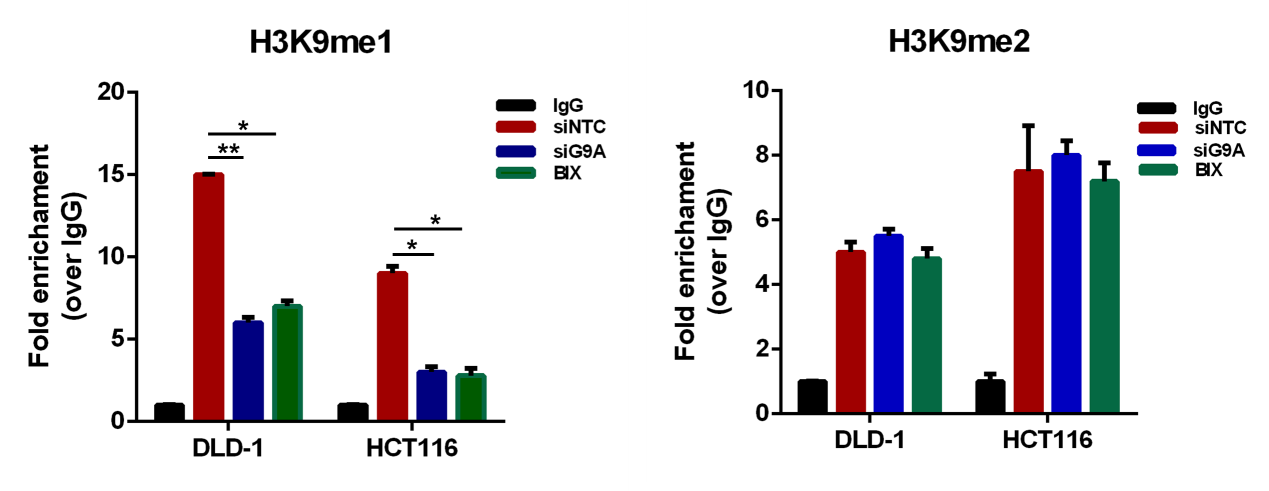


**Fig. S8 CHIP-qPCR analysis of H3K9me1 and H3K9me2 levels at the promoters of PSAT1 in HCT116 and DLD-1 after depletion of G9A by siRNA or BIX.**

**
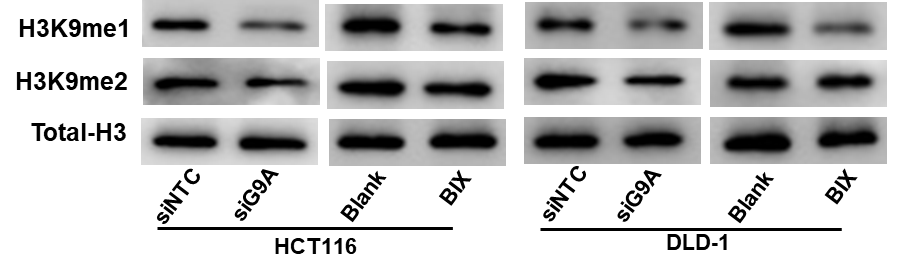
**

**Fig. S9 Levels of H3K9me1 and H3K9me2 proteins were analyzed using Western blot after depletion of G9A by siRNA or BIX.**


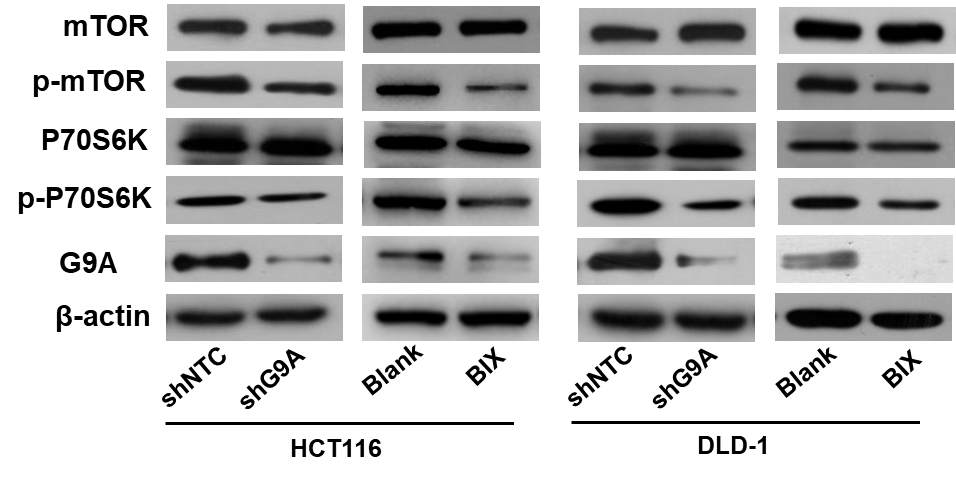


**Fig. S10 The protein expression levels of total mTOR, p-mTOR, total P70S6K and p-P70S6K were examined by western blot in HCT116 and DLD-1 cells after depletion of G9A by shRNA or BIX**


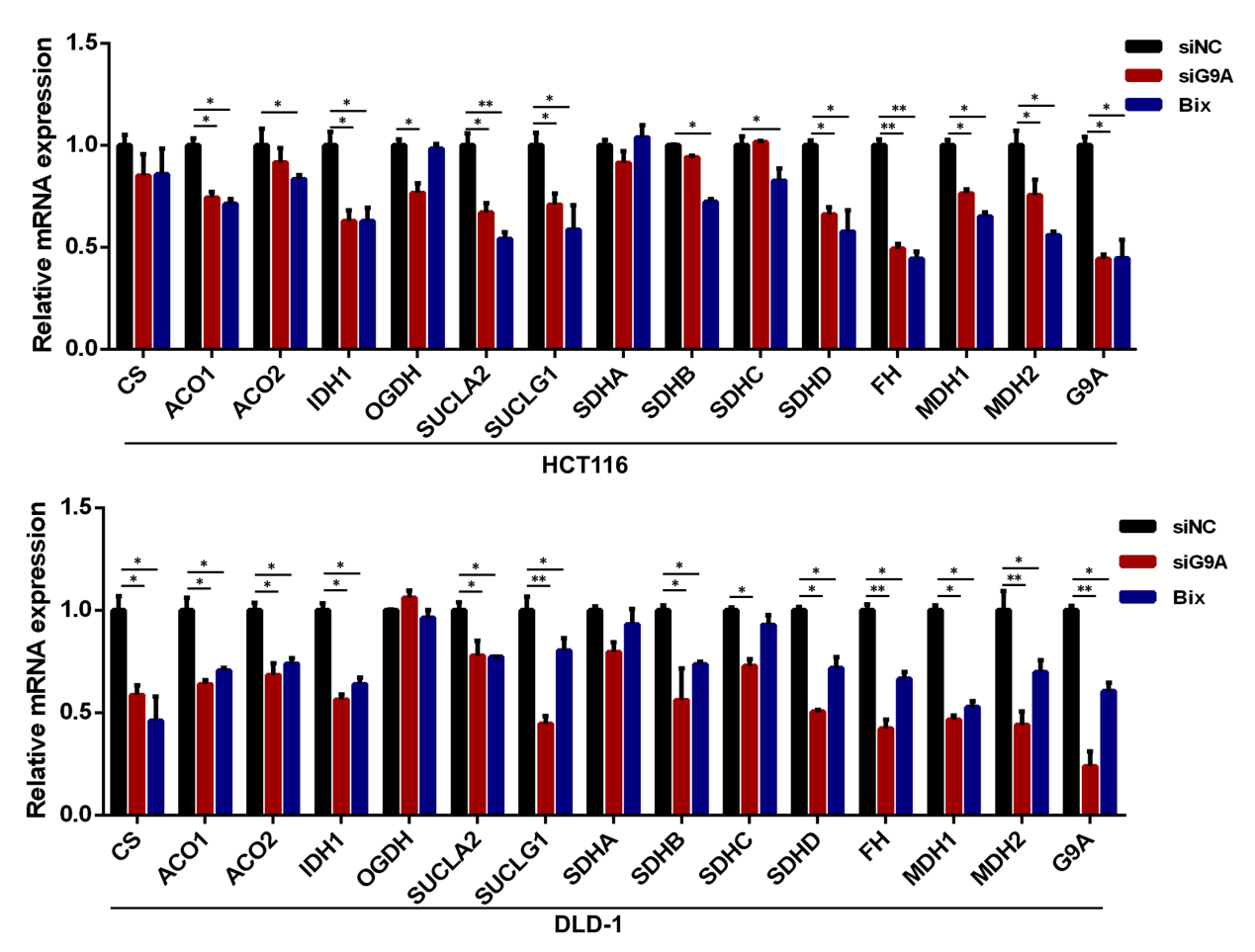


**Fig. S11 The mRNA levels of the TCA metabolic enzymes after depletion of G9A were examined by Real-Time PCR assay. (*p< 0.05, **p< 0.01).**

Abbreviations: CS:citrate synthase, ACO:aconitase, IDH:isocitrate dehydrogenase, OGDH:α-ketoglutarate dehydrogenase, SUCL: succinate CoA ligase, SDH:succinate dehydrogenase, FH:fumarate hydratase, MDH:malate dehydrogenase.
